# Analysis of a Series of 26 Cases With Prenatal Skeletal Dysplasia via Multiplatform Genetic Detection

**DOI:** 10.1002/mgg3.70062

**Published:** 2025-01-20

**Authors:** Li‐min Cui, Hua‐ying Hu, Xiao‐mei Zhai, Ming‐fei Qi, Yan‐ming Liu, Cong‐ying Han, Jing Zhang, Ming Shen, Yu‐lan Xiang, Wen‐qi Chen, Kai Yang, Dong‐liang Zhang, Huan‐xia Xing

**Affiliations:** ^1^ Prenatal Diagnosis Center Langfang Maternal and Child Health Care Hospital Langfang Hebei China; ^2^ Birth Defects Prevention and Control Technology Research Center Medical Innovation Research Division of Chinese PLA General Hospital Beijing China; ^3^ Prenatal Diagnosis Center, Shijiazhuang Obstetrics and Gynecology Hospital; Hebei Key Laboratory of Maternal and Fetal Medicine Shijiazhuang Key Laboratory of Reproductive Health Shijiazhuang Hebei China; ^4^ Department of Orthodontics, Beijing Stomatological Hospital, Capital Medical University School of Stomatology Capital Medical University Beijing China; ^5^ Prenatal Diagnosis Center, Beijing Obstetrics and Gynecology Hospital; Beijing Maternal and Child Healthcare Hospital Capital Medical University Beijing China

**Keywords:** chromosome microarray analysis, karyotyping, skeletal dysplasia, whole exome sequencing

## Abstract

**Background:**

Skeletal dysplasia (SD) represents a series of highly heterogeneous congenital genetic diseases affecting the human skeletal system. Refined genetic diagnosis is helpful for the accurate diagnosis and prognosis evaluation of SDs.

**Materials and Methods:**

In this study, we recruited 26 cases of SD and analyzed them with a designed sequential genetic detection. Chromosome karyotyping, microarray analysis (CMA), and whole exome sequencing (WES) techniques are performed as needed. Sanger sequencing and fluorescent quantitative PCR (QF‐PCR) were used as validation methods.

**Results:**

A total of 16 cases (61.5%, 16/26) received positive results at various levels of testing, including one trisomy 18, four copy number variations (CNVs), and 11 sequence variations. Additionally, four novel SD‐related sequence mutations were detected in this study.

**Conclusion:**

Our findings provide conclusive evidence for genetic counseling of corresponding families and expand the mutation spectrum of SD. In addition, this study demonstrates that a strategy sequentially including various genetic techniques contributes to the diagnosis of highly heterogeneous genetic disorders such as SD.

## Graphical abstract

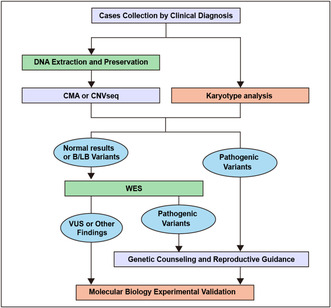

## 1 Introduction

Skeletal dysplasia (SD) represents a series of heterogeneous genetic disorders affecting approximately 2.3–4.5 in 10,000 births, which generally affects the growth, morphometry, and integrity of cartilage and/or bone (Orioli, Castilla, and Barbosa‐Neto 1986). SDs are individually rare, yet collectively comprise a large group of disorders ranging from mild anomalies to lethality (Schramm and Mommsen 2018; Krakow and Rimoin 2010). Skeletal involvement is also presented in hundreds of genetic syndromes (Offiah 2015). Since its first release, the SD nomenclature has been updated 10 times over the past 5 decades, and has now evolved to include 771 entries and 552 genes, as a dyadic naming system concerning both phenotypic features and genetic etiology (Unger et al. 2023). Overlapping phenotypes, the emergence of pathogenic genes and inheritance patterns among many SD subtypes make it more and more difficult for an accurate diagnosis.

Prenatal sonographic screening is still the indispensable first‐line method for SD screening, while advances in genetic techniques have made it possible to accurately type SD prenatally, providing solid clues for the prognosis and corresponding consultation of affected fetuses. Previous studies have used targeted exome sequencing for SD cohort detection (Bae et al. 2016; Zhou et al. 2018), but such schemes have been unable to cope with the rapidly expanding list of disease‐causing genes. In a previous pilot study of ours, trio WES (whole exome sequencing) had a 75% detection rate for eight SD cases with a normal karyotype and CMA (chromosome microarray analysis) results (Yang et al. 2019). A recent systematic review of ten studies concluded that WES had a pooled diagnostic yield of 69% for such cases (Tse et al. 2023). Considering cost and efficiency issues, a sequential prenatal genetic testing strategy covering karyotype, CMA, and NGS (next generation sequencing) is appropriate at present (Huang et al. 2023). The typical challenges in this field are improving detection levels and establishing associations between specific phenotypes and genotypes.

In the present study, we recruited 26 fetuses suspected of SDs based on prenatal ultrasonography and submitted them to a sequential genetic testing with the aim of identifying the causative variation. The relationship between causative variations and phenotypes of affected participants, as well as pregnancy management of corresponding families was also discussed.

## 2 Materials and Methods

### 2.1 Subjects and Clinical Evaluation

The present study was approved by the Ethics Committee of Langfang Maternal and Child Health Care Hospital (No. 2022‐KY‐003‐01). Informed consent was provided by all the participants. All procedures performed in the present study were in accordance with the Declaration of Helsinki 1964 and its later amendments or comparable ethical standards.

Inclusion criteria mainly rely on prenatal ultrasonography diagnosis and family history investigation of specific cases. We included cases of fetuses who exhibited SD throughout pregnancy and those who had a family history of SD. Sonographic diagnosis was performed in the second or third trimester. Detailed information, including maternal age, gestational weeks, obstetric history, and family history, was documented.

**FIGURE 1:**
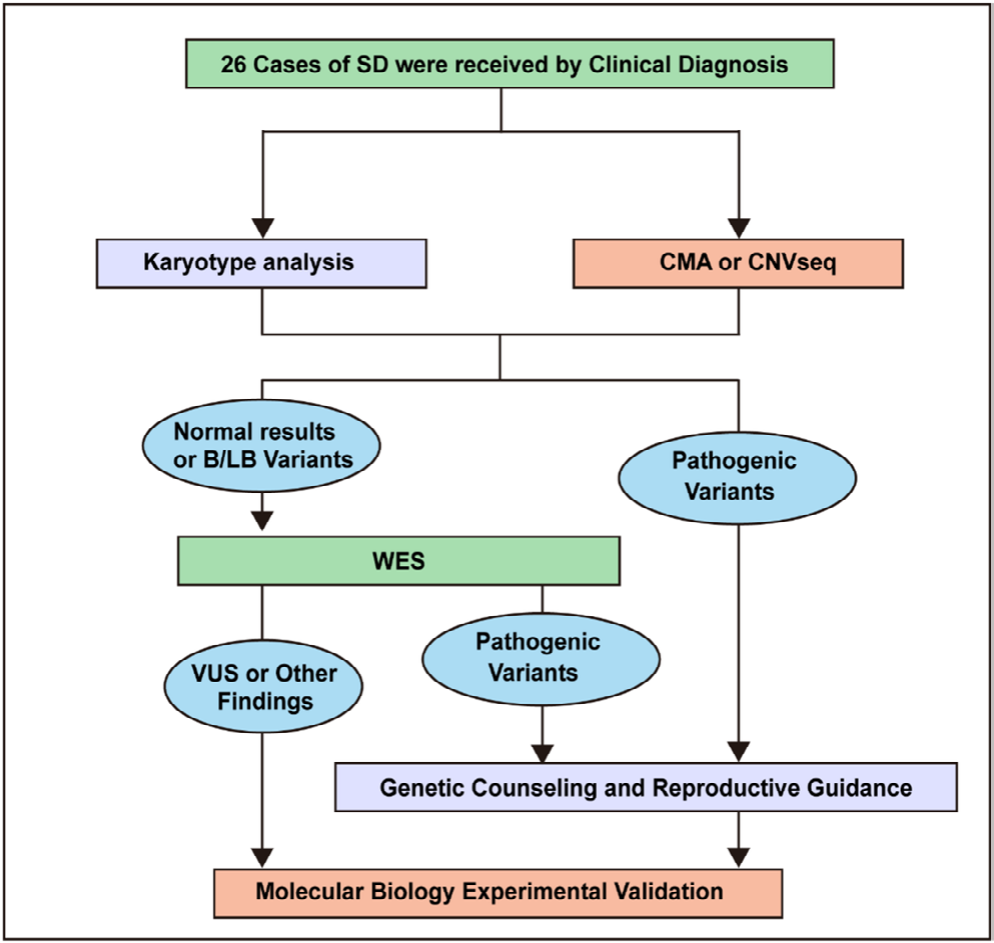
The main detection workflow of this study.

The main experimental workflow was demonstrated in Figure 1. After amniocentesis, chromosome karyotyping, DNA extraction of the corresponding samples, and the subsequent CMA (chromosome microarray analysis) were performed simultaneously. Performing the WES testing depends on whether the previous test yielded results as normal or benign/likely benign variations. Genetic counseling and reproduction guidance were carried out based on diagnostic variations at each level of genetic testing. For variants with unclear significance, it is recommended to conduct further functional verification experiments.

### 2.2 Chromosome Karyotyping

Fetal specimens including amniotic fluid (AF) or umbilical cord blood (UCB) were collected by routine procedure. Conventional chromosomal karyotyping by G‐binding was performed to detect overall chromosomal anomalies (Arsham, Barch, and Lawce 2017).

### 2.3 Chromosome Microarray Analysis (CMA)

Genomic DNA was extracted from fetal samples and their parents' PB samples using QIAamp DNA Midi Kit (Qiagen, Germany). As described in our previous study (Yang et al. 2022), CMA tests with CytoScan HD SNP‐array (Affymetrix Inc., USA) were carried out according to the manufacturer's manual workflow on all fetal specimens in order to investigate genomic CNVs with clinical significance.

### 2.4 Whole Exome Sequencing (WES)

WES was employed to detect the sequence variants in the probands' samples, as described in our previous study (Huang et al. 2021). Briefly, the target‐region sequences enrichment was performed using the Agilent Sure Select Human Exon Sequence Capture Kit (Agilent, USA). DNA libraries were tested by quantitative PCR, where the size, distribution, and concentration were determined using the Agilent Bioanalyzer 2100 (Agilent, USA). Along with ~150 bp pair‐end reads, the NovaSeq 6000 platform (Illumina Inc.) was used for sequencing of DNA with ~300 pM per sample with NovaSeq Reagent kit. Sequencing raw reads (quality level Q30% > 90%); and the quality criteria listed at https://www.illumina.com/science/technology/next‐generation‐sequencing/plan‐experiments/quality‐scores.html were aligned to the human reference genome (accession No. hg19/GRCh37) using the Burrows Wheeler Aligner tool, and PCR duplicates were removed using Picardv1.57. Variant calling was performed with the Verita Trekker Variants Detection system (v2.0; Berry Genomics, China) and Genome Analysis Tool Kit (https://software.broadinstitute.org/gatk/). Then, variants were annotated and interpreted using ANNOVAR (v2.0) (Wang, Li, and Hakonarson 2010) and Enliven Variants Annotation Interpretation systems (Berry Genomics), based on the common guidelines by ACMG (American College of Medical Genetics and Genomics) (Richards et al. 2015). To assist in the interpretation of variant pathogenicity, we referred to 3 frequency databases (ExAC_EAS, http://exac.broadinstitute.org; gnomAD_exome_EAS, http://gnomad.broadinstitute.org; 1000G_2015aug_eas, https://www.internationalgenome.org) and HGMD (Human Gene Mutation Database) pro v2019; Revel score (a combined method of pathogenicity prediction) (Ioannidis et al. 2016) and pLI score (representing the tolerance for truncating variants) were also employed.

Sanger sequencing was performed as a validation method with the 3500DX Genetic Analyzer (Applied Biosystems, Thermofisher, USA). Fluorescent quantitative PCR (QF‐PCR) was also conducted to validate the exonic copy number variants.

### 2.5 Conservatism Analysis

The evolutionary conservatism of all affected amino acid (AA) residues by corresponding missense variants was analyzed using the online tool, MEGA7 (http://www.megasoftware.net/previousVersions.php), with default parameters.

## 3 Results

### 3.1 Clinical Manifestation

A total of 26 cases with suspected in utero skeletal features were enrolled between July 2019 and May 2023. In the 26 recruited families, the average maternal age was 31.2 (ranging from 21 to 44), and the gestational weeks with initial SD diagnosis ranged from 13W5D to 30 W3D. Among these cases, 20 were sporadic and six had familial symptoms (Case 3, 9, 10, 12, 13, and 23) (see details in Table 1). All the couples claimed that they were non‐consanguineous.

**TABLE 1:** Clinical information of the 26 recruited cases.

Case No.	Maternal age (years)	Gestational age with initial diagnosis	Clinical history	Testing type	Pregnancy outcome according to follow‐up
1	31	24 W	G2P1. The first pregnancy was a delivery of twin girls with normal signs; the second pregnancy was aborted at the 31st week after ultrasonography showing short long bones at the 24th week and the 30th week.	KA + CMA + WES	Terminated
2	21	20 W3D	G2P1. The first pregnancy was induced abortion. The ultrasound result of the second pregnancy from 20th week indicated that the long bones of the fetus were progressively shorter referring to gestational age.	KA + CMA + WES	Terminated
3	28	28W5D	G1P0. The pregnant woman was 140 cm tall and suffered from congenital cataract. Ultrasound result showed that the fetal right lens echo was enhanced, the intestinal tube echo was enhanced, and the femur and humerus length values were lower than −2SD.	KA + CMA	Terminated
4	25	25W3D	G1P0. One femur of the fetus was bent, and the decision was made to induce labor after genetic testing.	KA + CMA + WES	Terminated
5	23	15W6D	G1P0. Bilateral fetal radius absence, right foot varus ventricular septal defect, bilateral choroid plexus cyst.	KA + CMA + WES	Terminated
6	35	13W5D	G2P0. Fetal meningocele, spina bifida, abnormal left hand posture, absence of left upper limb, left calf and foot.	KA + CMA	Terminated
7	33	22 W	G1P0. The long bones of the fetus' limbs were short.	KA + CMA	Terminated
8	37	25W4D	G2P0. In early pregnancy, NIPT suggested a partial increase in chromosome 16. The values of humerus and femur were lower than −2SD in the second trimester.	KA + CNVseq+WES	The fetus was born prematurely at 28 weeks and underwent cardiac catheter shunt surgery.
9	33	20 W	G2P1. The pregnant woman was 127 cm tall and had a daughter, aged 10, 122 cm tall. WES detected a heterozygous *FGFR3* mutation in both of them. This time, prenatal diagnosis was carried out to verify the situation of the fetus.	KA + CMA + WES	Terminated
10	27	/	G1P0. The pregnant woman presented with scoliosis. WES detected a heterozygous *MYH3* mutation in her. At 20th week, the fetus was asymptomatic. Prenatal diagnosis was conducted to verify whether the fetus carried the same mutation.	KA + CMA + WES	Continued to full‐term birth.
11	31	24 W2D	G1P0. The fetal femurs were curved and short. The parents were asymptomatic.	KA + CNVseq+WES	Terminated
12	33	30 W	G4P1. The pregnant woman was 150 cm tall and had metaphyseal dysplasia. She had a daughter, being 6 years old, 110 cm tall. The pregnant woman's sister was 140 cm tall. The fetus presented with long and short bones in late pregnancy.	KA + CMA + WES	Terminated
13	29	/	G2P1. The pregnant woman presented with short stature, left leg dysplasia, tibial pseudojoint, multiple subcutaneous nodules. Her father and sister were both short stature. She had a daughter, 4 years old, normal height.	KA + CMA + WES	Continued to full‐term birth.
14	44	24 W3D	G2P0. The overall fetal growth was delayed, and all the measured values were less than −2SD, and the length of humerus and femur was less than −4SD.	KA + CNVseq+WES	Terminated
15	38	29 W2D	G2P0. Fetal femur, humerus length less than −2SD, and left ventricular had stronger echos.	KA + CMA + WES	Terminated
16	39	29 W	G1P0. Fetal femur, humerus length less than −2SD.	KA + CMA + WES	Preterm delivery at 32 W6D, weight 1300 g, no apparent abnormality.
17	28	24 W5D	G1P0. The measurements of fetal femur coincided with the −2SD line.	KA + CMA + WES	Cesarean section at 38 weeks, no abnormality.
18	26	29 W	G1P0. The measured values of fetal humerus and femur were less than −4SD.	KA + CMA + WES	Pregnancy 38W2D vaginal delivery, no abnormalities.
19	34	26 W	G2P0. The long bones of the fetus were short and curved, the intervertebral distance of the spine was widened, and the nasal bone was abnormal.	KA + CNVseq+WES	Terminated
20	27	16 W	G1P0. The long bones of the fetus' limbs were severely short and curved.	KA + CMA + WES	Terminated
21	28	30 W	G1P0. The measured values of fetal humerus and femur were less than −2SD.	KA + CMA + WES	38W6D cesarean section, no abnormalities
22	37	32 W	G2P1. First pregnancy normal. This time, the fetal femur length was less than −2SD and the humerus length was less than −4SD.	KA + CMA + WES	Continued.
23	34	/	G2P1. The first child died of osteopetrosis at age 2 after birth, and a compound heterozygous variation in *SNX10* was identified; this time was for prenatal diagnosis for the 2nd pregnancy.	KA + CNVseq+WES	Continued.
24	25	21 W	G1P0. Fetal in utero fetal growth restriction; short and deformed limb bones.	KA + CMA + WES	Terminated
25	31	27 W	G2P0. The head circumference of the fetus was greater than 2SD, the long bones of the limbs were short, and the echo of both kidneys was enhanced.	KA + CMA + WES	Terminated
26	30	30 W3D	G1P0. Persistent left superior vena cava and coronary sinus dilatation; the proximal extremities of the long bones were shorter than the clinical gestational week.	KA + CMA	Terminated

Abbreviations: A, abortus; AF, amniotic fluid; CMA, chromosomal microarray analysis; CNVseq, copy number variation sequencing; D, days; G, gravida; KA, karyotype analysis; P, para; UCB, umbilical cord blood; W, weeks; WES, whole exome sequencing.

All the cases included in this study showed typical but various SD characteristics under ultrasound imaging tests, such as short stature, short long bone, long bone curvature, abnormal joint posture, etc. Individual cases were associated with indications in other systems. The detailed clinical manifestations and information of the 26 cases were listed in Table 1. The representative clinical images of affected fetuses were included in Figure S1 (Note: according to patients' discretion to privacy disclosure, not all clinical images of cases were included).

### 3.2 Genetic Findings

After sequential testing, diagnostic variations at various levels were detected in 16 cases (detection rate at 61.5%, 16/26).

**FIGURE 2:**
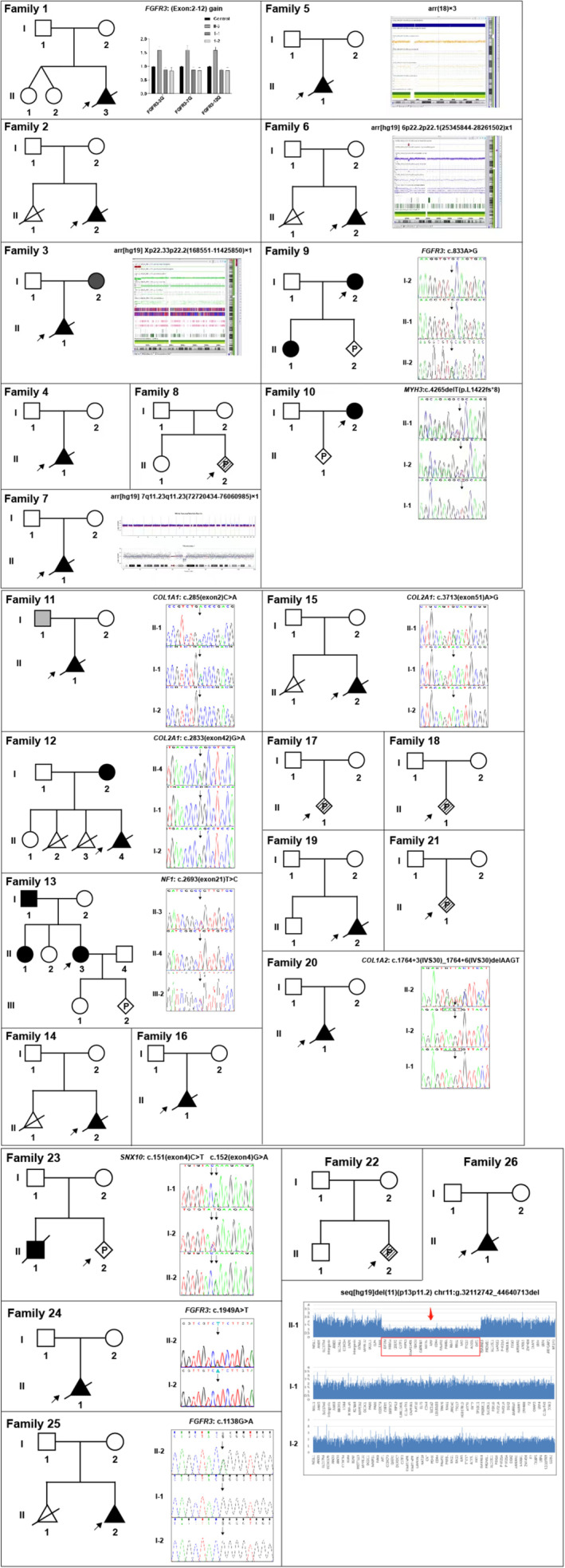
The pedigree diagrams and variants identified in this study. Part A, the pedigree diagrams and/or variants identified for Family 1–10. Part B, the pedigree diagrams and/or variants identified for Family 11–21. Part C, the pedigree diagrams and/or variants identified for Family 22–26. The triangle represents the fetus; diamond represents gender unknown; the black block represents affected; gray block represents carrier of variation but no clinical indications; the shaded lines represent clinical indications only; P stands for prenatal diagnosis.

To be specific, chromosomal abnormality was present in only one case, that is, Case 5 was found to have trisomy 18. Four cases harbored genomic copy number variations (CNVs), which were considered to be associated with their respective phenotypes (Case 3, 6, 7, and 26) (see details in Table 2 and Figure 2). Eleven cases carried sequence variations in various genes, including *FGFR3* (Case 1, 9, 24, and 25), *COL1A1* (Case 11), *MYH3* (Case 10), *COL2A1* (Case 12 and 15), *NF1* (Case 13), *COL1A2* (Case 20), and *SNX10* (Case 23). The detailed information of each sequence variant was included in Table 3. The Sanger sequencing or QF‐PCR results and the variant carrying status in each pedigree was demonstrated in Figure 2.

**TABLE 2:** Information on the copy number variations identified in this study.

Case No.	Genomic alteration	Size	Origin	Interpretation score	Rating
3	arr[hg19] Xp22.33p22.2(168,551‐11,425,850)×1	11.2 Mb	Mat	1A + 2A + 2G + 3C	P
6	arr[hg19] 6p22.2p22.1(25,345,844‐27,261,502)×1	2.9 Mb	de novo	1A + 2G + 3C	LP
7	arr[hg19]7q11.23q11.23(72,720,434‐76,060,985)×1	3.34 Mb	de novo	1A + 2A + 2G + 3C	P
26	arr[hg19]11p13p11.2(32,112,742‐44,640,713)×1	12.53 Mb	de novo	1A + 2A + 2B + 2G + 3C	P

**TABLE 3:** Information on the sequence variations identified in this study.

Case No.	Gene^a^	DNA sequence variation	Protein variation	Origin^a^	HGMD rating (dbSNP index)^a^	Frequency in databases^a^	Revel prediction^a^	Pathogenicity level (evidences)^a^
1	*FGFR3*	(Exon:2–12) gain	/	de novo	/	0; 0; 0	/	P
9	*FGFR3*	c.833A>G	p.Y278C	Mat	DM (rs121913115)	0; 0; 0	0.785	LP (PM1 + PM2‐Supporting +PP3‐Supporting+PP5‐Supporting)
10	*MYH3*	c.4265delT	p.L1422fs*8	Mat	/	0; 0; 0	/	LP (PVS1 + PM2‐Supporting)
11	*COL1A1*	c.285C>A	p.C95*	Pat	/	0; 0; 0	/	LP (PVS1 + PM2‐Supporting)
12	*COL2A1*	c.2833G>A	p.G945S	Mat	DM (rs886039542)	0; 0; 0	0.984	P (PS4‐Supporting+PM1 + PM2‐Supporting+PP2 + PP3 + PP4‐Strong)
13	*NF1*	c.2693T>C	p.L898P	Mat	DM (rs199474786)	0; 0; 0	0.959	P (PS4‐Supporting+PS2 + PM1+PM2‐Supporting+PP2 + PP3)
15	*COL2A1*	c.3713A>G	p.Y1238C	de novo	DM (rs199726428)	0; 0; 0	0.718	LP (PS2 + PM1 + PM2‐Supporting+PP2 + PP3)
20	*COL1A2*	c.1764 + 3_ c.1764 + 6del AAGT	/	de novo	/	0; 0; 0	/	LP (PS2 + PM2‐Supporting+PP3)
23	*SNX10*	c.151C>T	p.R51*	Mat	/(rs1353879401)	0; 0; 0.0001	/	LP (PVS1 + PM2‐Supporting)
		c.152G>A	p.R51Q	Pat	DM (rs398123011)	0; 0; 0	0.642	LP (PM2‐Supporting+PM3+PP1‐Strong+PP3)
24	*FGFR3*	c.1949A>T	p.K650M	de novo	DM (rs121913105)	0; 0; 0	0.832	P (PS3 + PM5‐Strong+PP1‐Strong+PM1 + PM2‐Supporting+PM6+PS4‐Moderate)
25	*FGFR3*	c.1138G>A	p.G380R	de novo	DM (rs28931614)	0; 0; 0	0.696	P (PS4‐VeryStrong+PS2‐VeryStrong+PS3 + PS1 + PM2‐Supporting)

^a^ Gene transcripts no.: *COL1A1*, NM_000088.3; *COL1A2*, NM_000089.3; *COL2A1*, NM_001844; *FGFR3*, NM_000142; *MYH3*, NM_002470; *NF1*, NM_001042492; *SNX10*, NM_013322; Pat, paternal; Mat, maternal; HGMD, Human Gene Mutation Database (Professional Version 2021.10); DM, disease‐causing mutation; dbSNP, https://www.ncbi.nlm.nih.gov/snp/; Three frequency databases: 1000g2015aug_eas (https://www.internationalgenome.org/); ExAC_EAS (http://exac.broadinstitute.org); gnomAD_exome_EAS (http://gnomad.broadinstitute.org/); Revel: An ensemble method for predicting the pathogenicity of missense variants on the basis of individual tools: MutPred, FATHMM, VEST, PolyPhen, SIFT, PROVEAN, MutationAssessor, MutationTaster, LRT, GERP, SiPhy, phyloP, and phastCons (https://doi.org/10.1016/j.ajhg.2016.08.016); Pathogenicity level rating: By ACMG (The American College of Medical Genetics and Genomics); P, pathogenic; LP, likely pathogenic; VUS, variants of unknown significance.

In regard to the four CNVs, three of them were de novo, and the rest one in Case 3 was of maternal origin (Table 2). In terms of the 11 cases with sequence variations, five probands carried de novo variants, while the other six were parentally inherited (Table 3 and Figure 2). Additionally, there were four sequence variants novelly reported in this study, namely *FGFR3*: (Exon:2–12) gain in Case 1, *MYH3*: c.4265delT(p.L1422fs*8) in Case 10, *COL1A1*: c.285C>A(p.C95*) in Case 11, and *COL1A2*: c.1764 + 3_c.1764 + 6delAAGT in Case 20.

### 3.3 Conservatism Analysis Results

**FIGURE 3:**
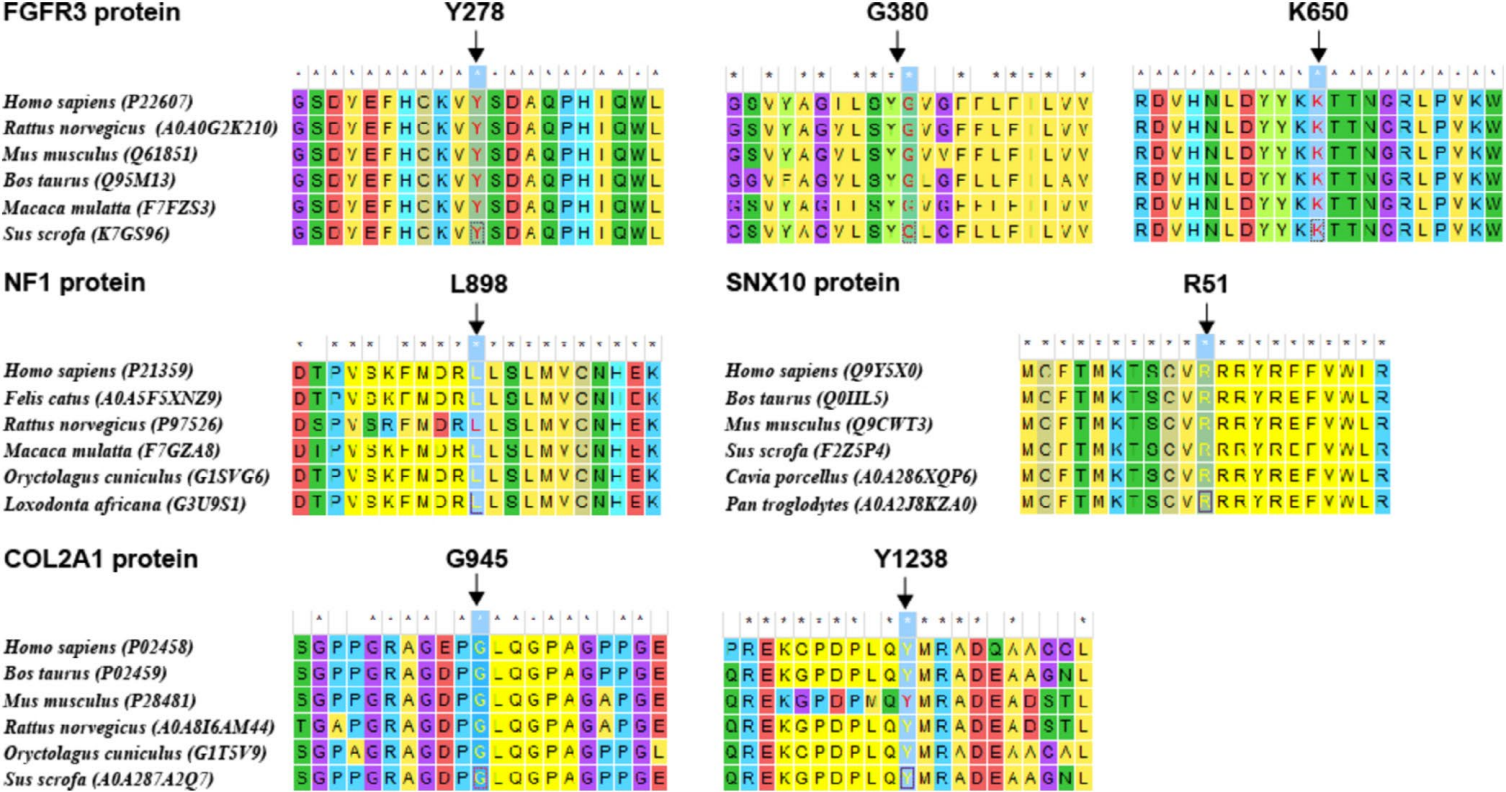
The conservativeness of the amino acid residues affected by the 7 detected missense variants across species.

A total of seven missense variants were detected in this study, namely *FGFR3*: c.833A>G(p.Y278C), *FGFR3*: c.1138G>A(p.G380R), *FGFR3*: c.1949A>T(p.K650M), *COL2A1*: c.2833G>A(p.G945S), *COL2A1*: c.3713A>G(p.Y1238C), *NF1*: c.2693T>C(p.L898P), and *SNX10*: c.152G>A(p.R51Q) (Table 3). MEGA 7 analysis demonstrated that the amino acid residues affected by these variants all maintained conservation across species, which indirectly supported the pathogenicity of each variant (Figure 3).

## 4 Discussion

Judging from the data of either perinatal or orthopedic medicine, SD occupies an important proportion in children with birth defects (Huang et al. 2023; Li et al. 2023). In the face of this daunting challenge, evolving genetic testing techniques have contributed to excellent capabilities of diagnosis and the discovery of new causative genes (Bae et al. 2016; Dennis, Greenhalgh‐Maychell, and Briggs 2021; Falardeau, Camurri, and Campeau 2017; de Pontual et al. 2011; Ng et al. 2010). Combining the detection efficiency of various techniques and economic considerations, a sequential detection strategy was proposed in this study. From the results of 26 SD cases, we achieved a positive detection rate of 61.5%, which is satisfactory and comparable to similar studies (Tse et al. 2023; Huang et al. 2023). Meanwhile, we identified four novel disease‐causing sequence variants that extended the mutation spectrum of the corresponding genes.

Although it is widely recognized that SDs are mostly monogenic disorders, aneuploid syndrome, the most common causes of birth defects, also has skeletal involvement (Krakow and Rimoin 2010; Cui et al. 2012). Therefore, as the basic means of prenatal genetic analysis, karyotyping can also elucidate some of SD cases. This study detected a case of trisomy‐18 induced SD by karyotyping, which was consistent with this situation. A large number of microdeletion or microduplication syndromes have been shown to be associated with SD, which can be detected by microarray or low‐coverage sequencing assays (Charan et al. 2014; de Wit et al. 2014; Flottmann et al. 2015; Holder‐Espinasse et al. 2019; Costantini et al. 2018). Our study identified four diagnostic CNVs (P/LP), among which one was maternally inherited, an 11.2 Mb heterozygous deletion at Xp22 in Case 3. The mother carrier herself also had short stature and congenital cataracts, and received a prosthetic eye transplant at the age of 18. This deletion segment contains 49 OMIM genes, among which *SHOX* (*312865) and *STS* (*300747) are associated two defined CNV syndromes, the Leri‐Weill dyschondrosteosis (#127300, LWD, pseudoautosomal, by *SHOX* deletion) (Binder and Rappold 1993) and steroid sulfatase deficiency (#308100, X‐linked ichthyosis, hemizygous, by *STS* deletion) (Richard 1993). LWD was clearly consistent with short limbs, while ocular symptoms may be associated with STS absence (Diociaiuti et al. 2019). The other three P/LP variations were all de novo. The 2.9 Mb deletion segment in Case 6 contains 79 OMIM genes, among which *H1‐4* (HIST1H1E, *142220) is associated with autosomal dominant Rahman syndrome (RS), characterized by intellectual disability, behavioral issues, skeletal involvement including scoliosis, talipes equinus and decreased bone mineral density (Burkardt and Tatton‐Brown 1993). The phenotype of Case 6 overlapped with previous RS reports to some extent, but the condition of limb mutilation might not be caused by this etiology, which is also worthy of further investigation. The 3.34 Mb deletion in Case 7 contains 43 coding genes, among which the *ELN* (*130160) gene is associated with supravalvular aortic stenosis (SVAS, #185500) characterized mainly by pulmonary valvular or peripheral arterial stenosis (Tassabehji et al. 1997), and also with cutis laxa‐1 (ADCL1, #123700) typified by loose and/or wrinkled skin that imparts a prematurely aged appearance (Tassabehji et al. 1998; Zhang et al. 1999). Meanwhile, this segment contains whole of the 7q11.23 recurrent Williams–Beuren syndrome (WBS) critical region. WBS may present with growth restriction and short stature, which is related to the imaging findings in this case (Morris 1993). The 12.53 Mb deletion in Case 26 contains 46 coding genes, among which *EXT2*, *ALX4*, and *WT1* are explicitly dosage sensitive (https://www.clinicalgenome.org/). The dbVar database included one case that overlapped with this deleted region, with clinical manifestations of intellectual deficiency, seizures, learning disabilities, and global development delay (https://www.ncbi.nlm.nih.gov/dbvar; case ID: nssv13652719). Among the above described genes, *EXT2* is related to SD, the mutation of which leads to multiple exostoses type II (EXT2; #133701) (Fusco et al. 2019); while *ALX4* mutation leads to parietal foramina‐2 (PFM2; #609597) (Altunoglu et al. 2014). Both disorders are inherited in an autosomal dominant pattern, resulting from the loss of function of the corresponding genes.

Subsequent WES identified the etiology of 13 cases, among which the variations in *FGFR3* (*134934) gene and collagen coding genes were the most common (8 cases in total), which was also consistent with previous studies (Zhang et al. 2021; Liu et al. 2019). The *FGFR3* variations in Cases 9, 24, and 25 were recurrent and shown to be causative for achondroplasia (ACH, #100800) (Heuertz et al. 2006), thanatophoric dysplasia type I (TD I, #187600) (Xue et al. 2014), and ACH (Foldynova‐Trantirkova, Wilcox, and Krejci 2012), respectively. The other variation in Case 1, (Exon:2–12) one copy gain, is novel and determined to be Pathogenic according to ACMG criteria. The intrauterine phenotype of this case was more similar to that of ACH. The collagen variations in Cases 11, 12, 15, and 20 are located in *COL1A1*, *COL2A1*, and *COL1A2*, respectively; among which, two were novel, namely *COL1A1*: c.285C>A(p.C95*) in Case 11 and *COL1A2*: c.1764 + 3_c.1764 + 6delAAGT in Case 20. Interestingly, the father carrier in Case 11 was asymptomatic, reflecting the intrafamilial phenotypic heterogeneity in OI (osteogenesis imperfecta) caused by certain mutations in type I collagen genes (Yang et al. 2022). The fetus in Case 20 had typical OI indications, suggesting that its variation may cause serious disruption of collagen I function. Mutations in type II collagen can also cause a broad range of collagenopathies; for example, *COL2A1* (*120140) mutations can lead to achondrogenesis type II (ACG2, #200610), Stickler syndrome (#108300), osteoarthritis with mild chondrodysplasia (OSCDP, #604864), Czech dysplasia (#609162), Kniest dysplasia (#156550), etc. The correlation between the variation of specific type and specific region of this gene and phenotype remains to be fully established. The patients in Case 12 did not have sensorial defects, suggesting that this variation mainly led to metaphyseal abnormalities, which was consistent with a previous report (Machol et al. 2017). The variation in Case 15 was de novo, and a previous report demonstrated that it could cause Stickler syndrome (Barat‐Houari et al. 2016).

The *MYH3* (*160720) gene is associated with a range of distal arthrogryposis disorders with mild to severe symptoms (Zhao et al. 2022). In a previous study, we diagnosed a case and discussed the genotype–phenotype association of this gene (Zhang et al. 2020). Here we identified a familial truncating variation in Case 10, with the mother carrier presenting with only moderate scoliosis. Therefore, the family made an informed choice to continue the pregnancy even though the prenatal diagnosis showed that the fetus also carried the variation and the fetus had no clear intrauterine indications. The *NF1* (*613113) gene is responsible for the neurofibromatosis type 1 (NF1, #162200), which also has skeletal involvement; and the variation in Case 13 was indicated to be pathogenic (Giugliano et al. 2019). This mother and some family members had skin and leg joint manifestations, and she had a normal daughter who did not carry this variation. Although the fetus also carried this variation, the family ultimately chose to keep it. In Case 23, a compound heterozygous variation was identified in *SNX10* (*614780) gene, which is responsible for the autosomal recessive osteopetrosis type 8 (OPTB8, #615085) (Aker et al. 2012). To date, fewer than 20 pathogenic variants of this gene have been identified (http://www.hgmd.cf.ac.uk/ac/index.php). The c.151C>T(p.R51*) variant was indexed in dbSNP database but not in HGMD, and its MAF (minor allele frequency) was 0.0001. The other variant, c.152G>A(p.R51Q), has already been reported to cause OPTB8 (Aker et al. 2012). The family's first child, who carried both variants, eventually developed osteopetrosis. Fortunately, prenatal diagnosis found that their existing fetus was a carrier of the c.152G>A variant as the father, so they ultimately decided to continue the pregnancy.

There were still ten cases in this study, although they were idiopathic and did not reach a positive conclusion, which is also worth for us to continue to follow up on their pregnancy status in the future and further analyze the test data after a period. We list the suspected SD associated VUS (variations with unknown significance) in these 10 cases in Data S1 to provide opportunities for further analysis and pathogenicity classification.

In terms of methodology selection and workflow creation, our study is only of some significance for reference. This is because, although the karyotype can detect balanced chromosomal structural variations and aneuploid chimeras that cannot be detected by other methods, the incidence of these is not high and their contribution to the pathogenesis of SD is limited. Therefore, whether to choose this technology in the regular process is also a discretionary choice. In addition, as WES technology has evolved, more and more studies have used it to conduct preliminary identification of CNVs. In addition, the cost of WGS (whole genome sequencing) is gradually decreasing, and it may integrally replace WES and other CNV detection methods in the future.

A major limitation of this study is that we did not obtain a large sample population out of a single causative gene to clearly establish specific genotypic and phenotypic associations. Although we strictly follow the principle of informed choice, the number of families who choose to terminate pregnancy in this study is still very high, which is largely related to our national situation of hardly accepting birth defects. This, coupled with the uncertainty and insignificance of prenatal phenotypes, made the process of analyzing and screening for genetic variants more difficult, which possibly had a certain impact on reporting outcomes.

In conclusion, we designed a relatively economical and effective testing scheme by using the current genetic techniques available in perinatal medicine. By using this protocol, 26 families with SD were diagnosed, and the positive rate was satisfactory. We found diagnostic variation at all levels, suggesting that each technique has its merits. We detected four novel variants, which enriched the mutant spectrum of SD. The heterogeneity of SD and the emergence of new causative genes still require continuous progress in genetic detection.

## Author Contributions

H.‐x.X., D.‐l.Z., K.Y., and L.‐m.C. conceived and designed the whole study; L.‐m.C., X.‐m.Z., M.‐f.Q., J.Z., Y.‐m.L., and W.‐q.C. recruited the cases and evaluated the clinical indications; H.‐y.H., K.Y., and C.‐y.H. performed the genetic experiments; L.‐m.C., M.S., and Y.‐l.X. analyzed the data; H.‐y.H. and K.Y. performed informatic analysis. L.‐m.C. wrote the original version of this manuscript, and K.Y. and H.‐x.X. reviewed and revised it. All authors approved the submission of this manuscript.

## Conflicts of Interest

The authors declare no conflicts of interest.

## Supporting information


Data S1.

## Data Availability

All relative data were shared in the article, and we have uploaded the sequencing results to figshare, a curated repository in general, at doi: 10.6084/m9.figshare.25027406.
