# Supplementary material for: Analysis of a Series of 26 Cases With Prenatal Skeletal Dysplasia via Multiplatform Genetic Detection
Source: Mol Genet Genomic Med. 2025 Jan 20;13(1):e70062. doi: 10.1002/mgg3.70062 (PMC11744476; doi:10.1002/mgg3.70062)
Supplement: Supplementary file 1 — Data S1. [file MGG3-13-e70062-s001.docx]

**Supplementary Material 1**

1. **Supplementary Figures**

**Figure S1. Imaging images and photos of some cases after induction of labor in this study; Note: The affected father's photo was shown in case 13.**


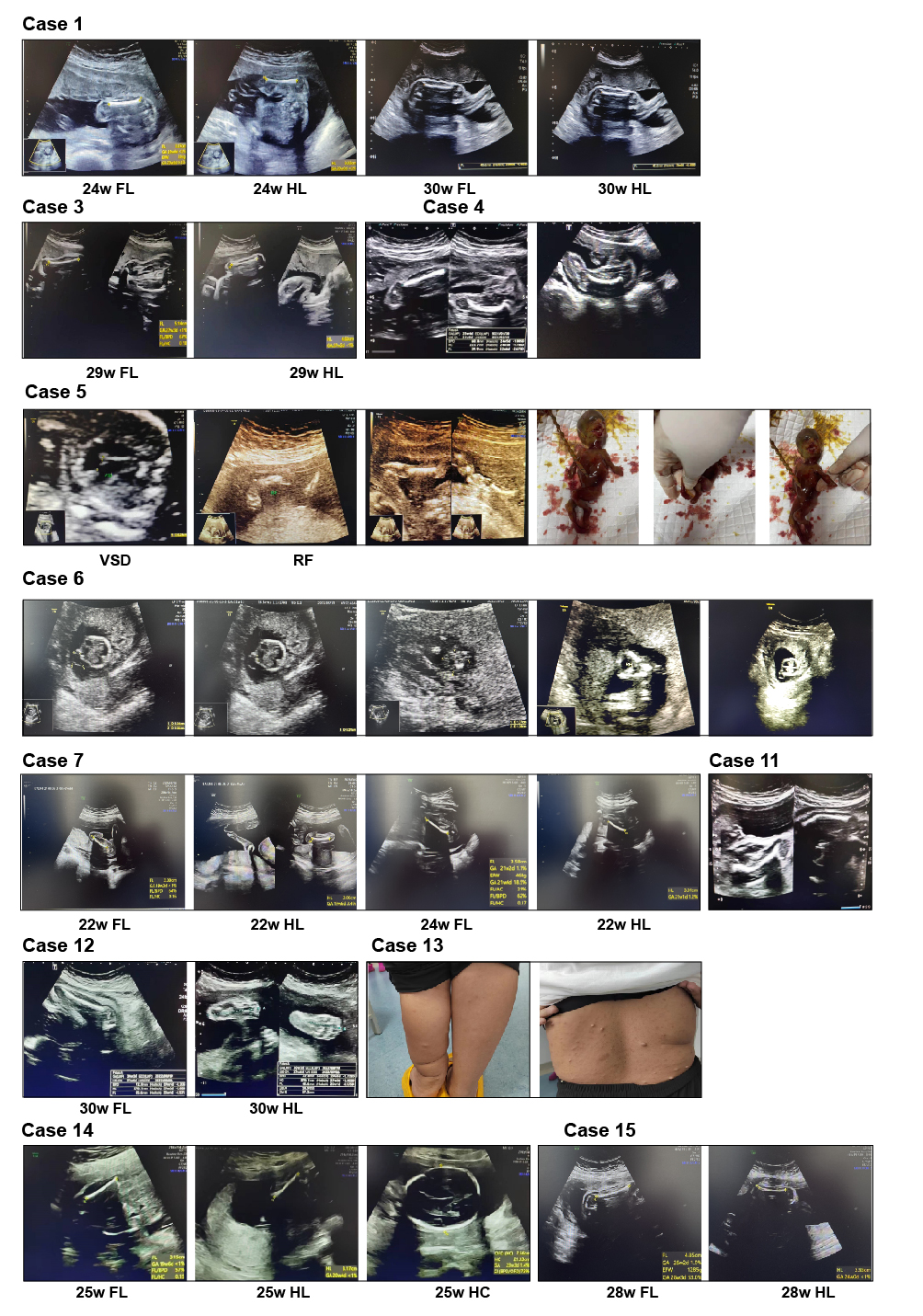


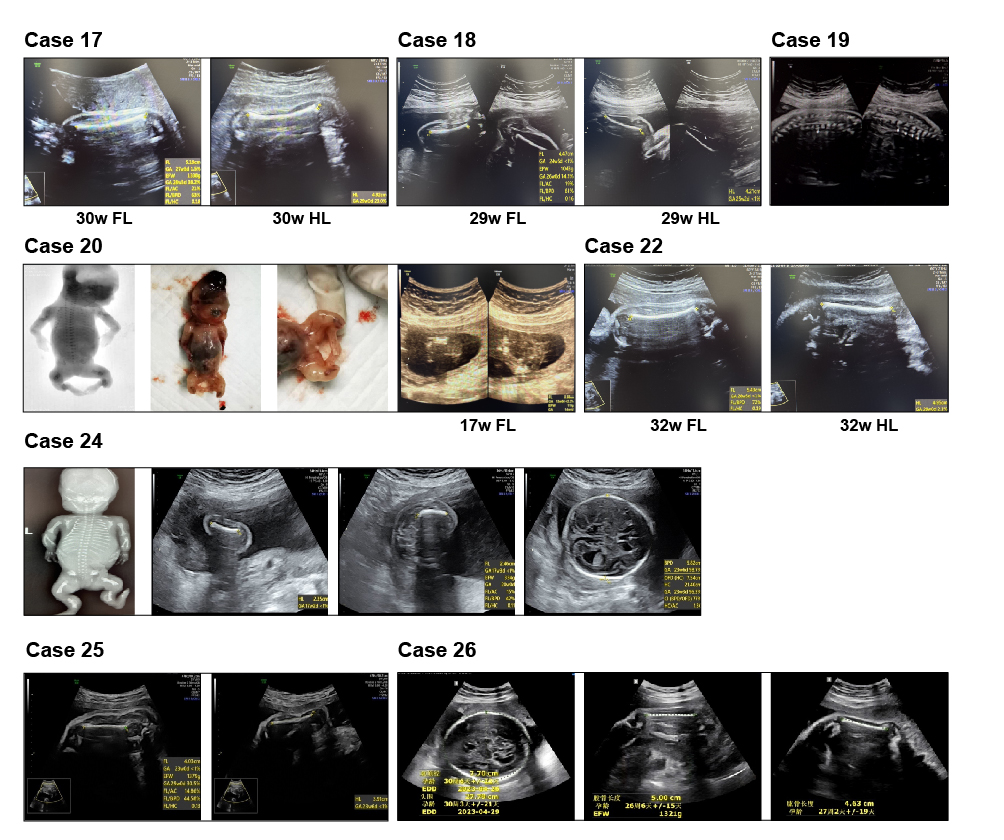


1. VUS list

(1) Case 2.

**CNV level:** arr[hg19] 17q25.3(80,374,898-81,041,823)x1. Size, 666.9Kb. This variant contains eight OMIM genes, including *WDR45B* (609226), *TBCD* (604649), *ZNF750* (610226), *B3GNTL1* (615337), etc. Homozygous mutations in the *WDR45B* gene are associated with autosomal recessive neurodevelopmental disorder with spastic quadriplegia and brain abnormalities with or without seizures. The clinical phenotypes include contracture, kyptoscoliosis, psychomotor development delay, intellectual impairment, inability to walk, aphasia, spastic paraplegia, brain hypoplasia. Homozygous or compound heterozygous mutation of *TBCD* gene and autosomal recessive Encephalopathy with brain atrophy and thin corpus callosum. Associated with brain atrophy and thin corpus callosum, clinical phenotypes include microcephaly, micrognathia, hypotonia, optic atrophy, face cleft, feeding difficulties, motor retardation, intellectual impairment, epilepsy, etc. Variants shorter than this segment deletion have been associated with global developmental delay (nsv3109701, heterozygote loss, uncertain significance), epilepsy (nsv817403, heterozygote loss, uncertain significance), Delayed puberty, intellectual impairment, plagiocephaly, torticollis (nsv2776337, heterozygote loss, uncertain significance), hypothyroidism, dystonia, moderate intellectual impairment (Patient: 278987, likely pathogenic) and other clinical phenotypic related cases were reported.

**Sequence level:** *DYNC2H1*(NM_001080463), c.6044G>A(p.R2015Q), heterozygous, paternally inherited. This variant is related to Short-rib thoracic dysplasia 3 with or without polydactyly (613091), but the disease is autosomal recessive (AR) inheritance. The absence of the trans allele variant makes the pathogenicity of the variant inconclusive.

(2) Case 4

**CNV level:** arr[hg19] Xq13.1(69,081,990-69,676,759)x3. Size: 594.7Kb. This variant contains eight OMIM genes, including *EDA* (300451), *IGBP1* (300139), etc. *EDA* gene mutation is associated with X-linked selective tooth agenesis-1 and other diseases. Clinical phenotypes include dental hypoplasia, endodontic disease, and hypokalemia. *IGBP1* gene mutation is associated with X -linked recessive corpus callosum, agenesis of, with mental retardation, ocular coloboma and micrognathia diseases, and the clinical phenotypes include short stature, giant stature, facial abnormalities, agenesis of the corpus callosum, and mental retardation.

**Sequence level:** *IGF2*(NM_001127598), c.97C>T(p.Q33*), heterozygous, paternally inherited. This gene has an imprinting effect, and paternal allele mutations may cause diseases such as SILVER-RUSSELL syndrome type 1 (180860) and SILVER-RUSSELL syndrome type 3 (616489). These diseases are associated with symptoms such as being small for gestational age and delayed bone maturation. The father of the subject carries the same mutation but the phenotype is normal, so it is necessary to further check the paternal phenotype, and the family verification of the proband's paternal grandparents can be added to determine the source of the variation of the paternal heterozygous site. If it comes from the grandmother, it can explain the normal phenotype of the father.

*COL1A2*(NM_000089), c.2565+4(IVS40)A>T, heterozygous, paternally inherited. The disease caused by this gene mutation is osteogenesis imperfecta (617268, 259420, 166220), which is consistent with the symptoms of femoral curvature and small for gestational age. The father had the same mutation but was asymptomatic, as has often been the case in previous reports of the disease. Further detection of cDNA levels may help determine pathogenicity.

(3) Case 8

**CNV level:** None.

**Sequence level:** None.

(4) Case 14

**CNV level:** None.

**Sequence level:** *OFD1*(NM_001330210), c.860T>C(p.L287P), hemizygous, maternally inherited. This gene is associated with orofaciodigital syndrome I (OFDS1, 311200) and Joubert syndrome type 10 (300804). Among them, OFDS1 has some skeletal system phenotypes, such as short stature and bone dysplasia, which may be related to the patient in this case. However, given the short stature of both parents, it is impossible to say for sure.

(5) Case 16

**CNV level:** None.

**Sequence level:** None.

(6) Case 17

**CNV level:** None.

**Sequence level:** None.

(7) Case 18

**CNV level:** None.

**Sequence level:** *COL1A1*(NM_000088), c.858+24(IVS12)G>A, heterozygous, paternally inherited. *COL1A1* mutations are associated with a series of developmental diseases, including osteogenesis imperfecta (166210, 259420, 166220, 166200, 166710). The father had the same mutation but was asymptomatic, as has often been the case in previous reports of the disease. Further detection of cDNA levels may help determine pathogenicity.

(8) Case 19

**CNV level:** None.

**Sequence level:** *RUNX2*(NM_001024630), c.1259C>T(p.T420I), heterozygous, paternally inherited. Mutations in this gene have been linked to two diseases with chromosomal dominant (AD) pattern, cleidocranial dysplasia (119600) and Metaphyseal dysplasia with maxillary hypoplasia with or without brachydactyly (156510). These two diseases are typical congenital skeletal conditions, both have short stature, epiphyseal dysplasia and other common characteristics. The father of the fetus carries the same variation, reducing the pathogenicity level of it. More experimental evidence may help interpret it further.

(9) Case 21

**CNV level:** None.

**Sequence level:** *NPR2*(NM_003995), c.2966G>A(p.R989Q), heterozygous, maternally inherited. Mutations in this gene are associated with two autosomal dominant disorders, epiphyseal chondrodysplasia, Miura type (615923) and short stature with nonspecific skeletal abnormalities (616255). The indications of the former are mainly tall stature, osteopenia and epiphyseal dysplasia, which are not consistent with this case. The latter is mainly characterized by delayed bone maturation and low body growth ratio, which may be related to this case. Maternal transmission reduces the variant's pathogenicity, making more experimental evidence necessary.

(10) Case 22

**CNV level:** None.

**Sequence level:** None.
